# A Novel Phenotype of Nonsteroidal Anti-Inflammatory Drug Hypersensitivity *The High-Risk Patient*

**DOI:** 10.1097/WOX.0b013e3181971b89

**Published:** 2009-02-15

**Authors:** Mario Sánchez-Borges, Arnaldo Capriles-Hulett, Fernan Caballero-Fonseca

**Affiliations:** 1 Allergy Department, Clínica El Avila, P. O. B. A. Internacional No. 635, Miami FL 33102-5255, Caracas, Venezuela; 2 Allergy and Immunology Department, Centro Médico-Docente La Trinidad, P. O. B. A. Internacional No. 635, Miami FL 33102-5255, Caracas, Venezuela; 3 Allergy Department, Centro Médico de Caracas, Caracas, Venezuela

**Keywords:** aspirin, angioedema, cyclooxygenases, NSAIDs, urticaria

## Abstract

**Background:**

Some nonsteroidal anti-inflammatory drug (NSAID)-hypersensitive patients develop adverse reactions when challenged with weak cyclooxygenase 1 (COX-1) inhibitors.

**Objectives:**

To investigate the prevalence and clinical features of this high-risk population.

**Materials and methods:**

Patients from 2 outpatient allergy clinics consulting between October 2005 and October 2007 because of adverse reactions to classic NSAIDs were submitted to confirmatory double-blind oral challenges with the suspected NSAID and with acetaminophen, preferential and/or specific COX-2 inhibitors. Patients were then classified as low-risk and high-risk groups according to the results of provocation tests.

**Results:**

Three hundred three patients were studied: 179 (59.0%) were tolerant to acetaminophen and the selective COX-2 inhibitors (low-risk group), whereas 124 (40.9%) developed reactions to at least one of the ''low COX-1 inhibitors'' (high-risk group). No distinctive demographic or clinical characteristics were present when both groups of patients were compared.

**Conclusions:**

A large proportion of patients sensitive to classic NSAIDs cannot tolerate the weak COX-1 inhibitors. Oral challenges should be performed by trained specialists to advise these patients about the use of NSAIDs.

## 

Hypersensitivity reactions to aspirin and nonsteroidal anti-inflammatory drugs (NSAIDs) are common in the population, and they are observed more often in young atopic individuals [1]. Different clinical patterns of reactions involving the skin, respiratory tract, or generalized have been described, and 4 forms of clinical presentation have been proposed: (1) respiratory (aspirin-exacerbated respiratory disease), (2) cutaneous (urticaria and angioedema), (3) mixed, and (4) systemic (anaphylaxis) [2].

Because many patients will react to NSAIDs of diverse chemical composition (cross reactions), it is unlikely that most of these reactions are mediated by specific immunologic mechanisms and it has been postulated that they are caused by inhibition of the isoenzyme cyclooxygenase 1 (COX-1), leading to excessive production of cysteinyl leukotrienes and a decreased synthesis of prostaglandin E2 [3,4]. In consequence, the new NSAIDs that selectively inhibit COX-2 and purportedly do not inhibit COX-1 were tested in NSAID-sensitive patients, and it was observed that preferential and specific COX-2 inhibitors were tolerated by most of them [5].

There is, however, a subset of patients who develop adverse reactions when challenged with conventional doses of COX-2 inhibitors [6-9]. Matucci et al[10] recently proposed that patients reacting to "low COX inhibitors" such as acetaminophen and nimesulide should be designated as a high-risk population of NSAID-hypersensitive patients. The present investigation was performed to determine the prevalence and clinical characteristics of this subpopulation at high risk of reactions to low COX-1 inhibitors.

## Materials and methods

All patients attending 2 outpatient allergy clinics in Caracas between October 2005 and October 2007 and complaining of urticaria, angioedema, asthma, or anaphylaxis occurring after taking NSAIDs were included in the study. Information on age, sex, history of allergic diseases, and drugs provoking the symptoms was obtained by direct patient questioning. After signing informed consent forms, they were submitted to double-blind placebo-controlled oral challenges with classic and selective NSAIDs[8] and to skin prick tests with inhalant allergens (ALK Abelló, Madrid, Spain).

Oral challenges were performed by means of a double-masked protocol. The drugs or placebo, given on different days, were concealed in identical opaque capsules, and half doses were administered 1 hour apart, with 3 hours of observation in the hospital and a telephone recall 24 hours later. Vital signs and pulmonary function (forced expiratory volume in 1 second, forced vital capacity, forced expiratory flow between 25% and 75%, and peak expiratory flow) were monitored at baseline and hourly for 3 hours, and the skin, nose, eyes, and thorax were examined at the same time intervals. The presence of breathlessness, cough, wheezing, dysphonia, nasal or ocular itching, sneezing, rhinorrhea, nasal obstruction, and conjunctival erythema was specifically investigated at every hourly physical examination. For urticaria and angioedema, the percentage of skin involved was calculated as follows: head and neck, 30%; chest, 20%; abdomen, 20%; upper limbs, 15%; and lower limbs, 15%. The test result was regarded as positive for urticaria or angioedema if involvement of 20% or more of the body surface area was present; and for respiratory reactions, if symptoms or signs appeared or a decrease in forced expiratory volume in 1 second greater than 20% of the basal condition was detected. Patients were challenged during remission of the urticaria or angioedema and with antihistamines and leukotriene modifiers withheld for at least 96 hours before testing. Corticosteroids were omitted 1 month before testing. Maximal challenge doses were: acetaminophen 500 mg, nimesulide 100 mg, meloxicam 15 mg, celecoxib 200 mg, rofecoxib 50 mg, etoricoxib 120 mg, and valdecoxib 40 mg. The protocol was approved by the institutional review boards of the participating institutions. The pharmacological and clinical pattern of the reactions was established as previously described [2].

### Statistical Analysis

Mean values were compared using Student *t *test. Proportions were compared using Fisher exact test.

## Results

**Figure 1:**
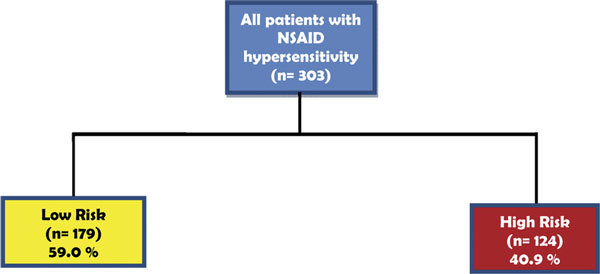
**Patients with NSAID hypersensitivity classified according to the risk of reactions to low COX inhibitors**.

**Figure 2:**
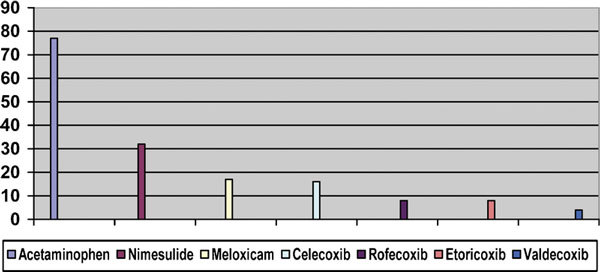
**Frequency of reactions to low COX inhibitors in 124 patients with a high risk of reactions to NSAIDs**.

During the period of the study, 303 patients with challenge-confirmed NSAID hypersensitivity reactions were observed. One hundred seventy-nine (59.0%) reacted to classic NSAIDs but tolerated acetaminophen and one or more selective or specific COX-2 inhibitors (low-risk group). One hundred twenty-four patients (40.9%) were classified as the high-risk group based on positive oral challenges to acetaminophen and selective or specific COX-2 inhibitors (Figure 1). Table 1 shows the demographic and clinical characteristics of the 2 groups of patients. Drugs responsible for reactions to low COX inhibitors observed in the high-risk group are shown in Figure 2, where it can be observed that the order of frequency of reactions was: acetaminophen, nimesulide, meloxicam, celecoxib, rofecoxib, etoricoxib, and valdecoxib. It must be mentioned, however, that the number of challenges was not the same for all the selective drugs, and therefore these data must be interpreted with caution.

**Table 1:** Demographic and Clinical Features of Patients With Low and High Risks for NSAID Hypersensitivity

	Low-Risk Group	High-Risk Group	*P*
n	179	124	--
Age ± SD (range), y	30.6 ± 12.8 (9-69)	32.7 ± 14.5 (10-90)	0.18
Sex, n (%)			
Female	120 (67.0)	93 (75.0)	--
Male	59 (32.9)	31.0 (25.0)	0.1
Atopic disease, n (%)			
Any atopic disease	162 (90.5)	104 (83.8)	--
Rhinitis	117 (65.3)	76 (61.2)	--
Rhinitis and asthma	41 (22.9)	26 (20.9)	--
Asthma	2 (1.1)	1 (0.8)	--
Rhinitis, asthma, and dermatitis	2 (1.1)	0 (0)	--
Rhinitis and dermatitis	0 (0)	1 (0.8)	--
None	17 (9.4)	20 (16.1)	0.1
Pharmacological pattern, n (%)			
Cross reactor	146 (81.5)	92 (74.1)	--
Single reactor	33 (18.4)	32 (25.8)	0.1
Clinical pattern, n (%)			
Cutaneous	99 (55.3)	61 (49.1)	0.3
Mixed	79 (44.1)	62 (50.0)	0.3
Systemic	1 (0.5)	1 (0.8)	0.6
Immediate-type skin tests, +/n (%)	148/173 (85.5)	97/105 (92.3)	0.1

A case report is included to illustrate the clinical presentation of a patient of the high-risk subset.

### Case Report

S.S.F. is a 66-year-old female patient who complained of eyelid angioedema, ocular itching, and facial and cervical erythema that occurred into the first 30 minutes after receiving various analgesics, including aspirin, pyrazolone, and ketoprofen. According to the patterns previously proposed, this patient was pharmacologically classified as cross-reactor and clinically, because of the presence of cutaneous and ocular symptoms, as mixed (blended). There was no history of other allergic or atopic diseases, and immediate-type skin hypersensitivity (prick) tests with 20 inhalant and 21 food allergens were negative. Double-blind oral provocation test results were as follows:

#### Meloxicam

One hour after receiving 7.5 mg of meloxicam, eyelid edema, breathlessness, dysphonia, and a sensation of laryngeal swelling were reported by the patient. Treatment with epinephrine (1:1000) 0.3 mL subcutaneously and chlorphenir-amine 100 mg intravenously were administered. The patient was discharged after total recovery 2 hours later.

#### Celecoxib

**Figure 3:**
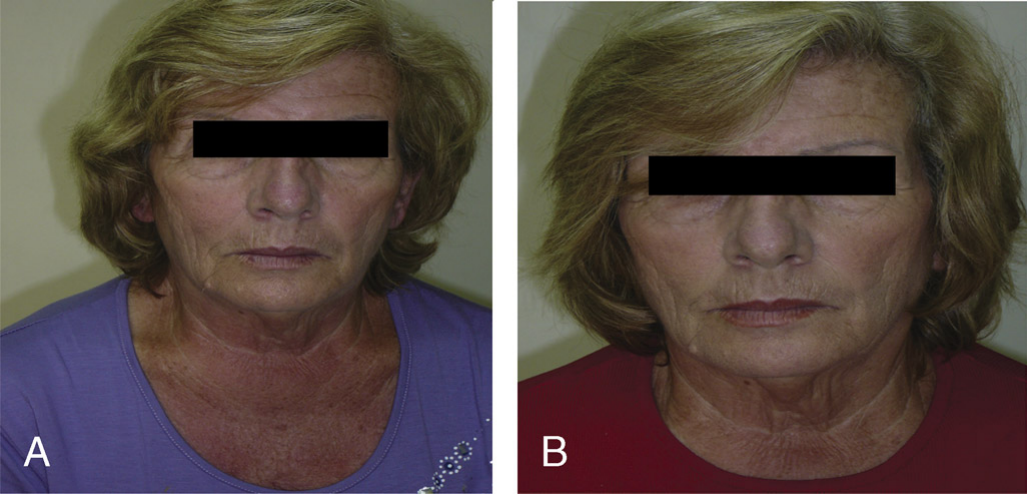
**A 66-year-old female NSAID-sensitive patient after oral challenge with (A) celecoxib and (B) nimesulide**.

Thirty minutes after a 100-mg provocative dose of celecoxib, an itchy erythematous rash was observed in the face, neck, and chest (Figure 3A). Oral chlorpheniramine 8 mg and montelukast 10 mg induced the disappearance of the rash in approximately 30 minutes.

#### Nimesulide

A challenge with 100 mg was tolerated (Figure 3B).

## Discussion

A subset of patients with hypersensitivity to NSAIDs cannot tolerate drugs that weakly inhibit COX-1. The reasons for these reactions are presently not clear, and this study intended to investigate the proportion of NSAID-sensitive patients who show an increased risk of reactions to acet-aminophen and selective COX-2 inhibitors, the high-risk subpopulation of individuals proposed by Matucci et al,[10] and to determine the clinical features that could help identify these patients.

As shown in Table 1, there were no distinctive clinical characteristics permitting to distinguish among low-risk and high-risk patients, including age, sex, presence of atopic diseases, positivity of immediate-type skin hypersensitivity tests with inhalant allergens, and pharmacological or clinical pattern.

The proportion of patients who reacted to weak COX inhibitors in this series was 40.9%, a considerable and important figure. Interestingly, among low COX inhibitors, acetaminophen was most commonly incriminated, followed by nimesulide, meloxicam, and less frequently, the coxibs, the more selective drugs sparing COX-1 (Figure 2). This is in agreement with the rates of inhibition of COX-1 in vitro,[11] with the exception of acetaminophen that inhibits another isoenzyme, COX-3, which is closely related to COX-1 [12-14].

The management of pain and inflammation in low-risk patients was accomplished with selective NSAIDs according to the results of oral challenges. In general, these patients continue to tolerate treatment with low COX inhibitors, although it has been occasionally reported that some patients will develop later on reactions with drugs that had been tolerated during controlled challenges [15]. In high-risk patients, such as the one described in this article, the management is more difficult, and in many of them, it is limited to the use of opioids for postsurgical pain and alternative analgesic methods such as acupuncture and others. Additional research is needed to offer new therapies to these patients with a high risk of reactions to NSAIDs.
